# Diastolic dysfunction in primary care: medical and nursing interlocutions

**DOI:** 10.1590/0034-7167-2025-0005

**Published:** 2025-12-08

**Authors:** Adson Renato Leite, Aretha Pereira de Oliveira, Dalmo Valério Machado de Lima, Maria Luiza Garcia Rosa, Antônio José Lagoeiro Jorge

**Affiliations:** I Universidade Federal Fluminense. Niterói, RJ, Brazil; II National Cancer Institute. Rio de Janeiro, RJ, Brazil

**Keywords:** Heart Failure, Echocardiography, Primary Health Care, Survival Analysis, Nursing Diagnosis., Insuficiência Cardíaca, Ecocardiografia, Atenção Primária à, Saúde, Análise de Sobrevida, Diagnóstico de Enfermagem., Insuficiencia Cardíaca, Ecocardiografía, Atención Primaria de Salud, Análisis de Supervivencia, Diagnóstico de Enfermería.

## Abstract

**Objectives:**

To evaluate the ratio between early and late mitral filling velocities (E/A) as a prognostic factor for hospitalizations due to cardiovascular causes and mortality in primary care; to identify the main NANDA-I nursing diagnoses, interventions, and outcomes associated with diastolic dysfunction.

**Method:**

633 individuals were recruited for evaluation of the E/A ratio and characterization of diastolic dysfunction. Multivariate Cox regression with Schoenfeld residual analysis, survival analysis, and hazard ratio estimation were performed.

**Results:**

After six years, crude hazard ratios of 3.47, 3.60, 3.02, and 0.41 were identified for classes II and III diastolic dysfunction at cut-off points 1 and 2, respectively (p<0.01). Nursing diagnoses of Activity Intolerance, Anxiety, Fatigue, and Deficient Knowledge should be prioritized.

**Conclusion:**

The E/A ratio showed an altered pattern in most participants without a diagnosis of heart failure, which may indicate a subclinical form of the condition.

## RESUMO

**Objetivos:**

Avaliar a razão entre as velocidades de enchimento mitral precoce e tardia (E/A) como prognóstico para hospitalizações por causas cardiovasculares e mortalidade na atenção primária; identificar os principais diagnósticos de enfermagem NANDA-I, intervenções e resultados associados à disfunção diastólica.

**Método:**

633 indivíduos foram recrutados para avaliação da razão E/A e caracterização da disfunção diastólica. Foram realizadas regressão de Cox multivariada com análise dos resíduos de Schoenfeld, análise de sobrevivência e estimativa da razão de risco (hazard ratio).

**Resultados:**

Após seis anos, foram identificadas razões de risco brutas de 3,47; 3,60; 3,02 e 0,41 para as classes II e III de disfunção diastólica nos pontos de corte 1 e 2, respectivamente (p<0,01). Diagnósticos de enfermagem Intolerância à Atividade, Ansiedade, Fadiga e Conhecimento Deficiente devem ser priorizados.

**Conclusão:**

A razão E/A apresentou padrão alterado na maioria dos participantes sem diagnóstico de insuficiência cardíaca, podendo indicar uma forma subclínica da condição.

## RESUMEN

**Objetivos:**

Evaluar la relación entre las velocidades de llenado mitral temprano y tardío (E/A) como pronóstico de hospitalizaciones por causas cardiovasculares y mortalidad en atención primaria; identificar los principales diagnósticos de enfermería NANDA-I, intervenciones y resultados asociados a la disfunción diastólica.

**Método:**

633 individuos fueran reclutados para evaluar la relación E/A y caracterizar la disfunción diastólica. Se realizaron regresiones de Cox multivariadas con análisis de residuos de Schoenfeld, análisis de supervivencia y estimación de la razón de riesgo (hazard ratio).

**Resultados:**

Después de seis años, se identificaron razones de riesgo brutas de 3,47; 3,60; 3,02 y 0,41 para las clases II y III de disfunción diastólica en los puntos de corte 1 y 2, respectivamente (p<0,01). Los diagnósticos de enfermería Intolerancia a la actividad, Ansiedad, Fatiga y Conocimiento deficiente deben ser priorizados.

**Conclusión:**

La relación E/A presentó un patrón alterado en la mayoría de los participantes sin diagnóstico de insuficiencia cardíaca, lo que podría indicar una forma subclínica de la afección.

## INTRODUCTION

Heart failure (HF) represents the final stage of most disorders and diseases affecting the cardiovascular system and is regarded as a major clinical and epidemiological healthcare challenge^(1)^. Population aging, combined with the rising burden of cardiovascular diseases (CVDs) and risk factors in developing countries, has led to an increase in the incidence and prevalence of HF, underscoring the importance of studies focused on its prognosis^(2)^. Historically, the diagnosis and classification of HF have been based on echocardiographic measurements, as recommended by guidelines in several countries since the 1990s^(3,4)^.

Asymptomatic cardiac dysfunction - whether predominantly systolic or diastolic - is highly prevalent among adults over the age of 45. It represents a preliminary stage of HF that may naturally progress to its clinical form. However, if identified early and treated appropriately, recovery of cardiac function is possible^(5)^. Beyond its diagnostic utility, echocardiography is used to classify HF phenotypes, determine its etiology, guide therapy, monitor treatment response, and establish prognosis^(6)^. Despite its clinical relevance, the use of echocardiography in primary care remains limited and often inaccessible. This highlights the need for innovative strategies to enable its cost-effective expansion. Underutilization of echocardiography hampers our understanding of echocardiographic predictors of morbidity and mortality in the general population^(7)^.

Diastolic dysfunction (DD) is defined by impaired relaxation and filling of the left ventricle, typically with preserved ejection fraction. Although often associated with hospitalized patients, DD is also prevalent in non-hospitalized populations, particularly among individuals with cardiovascular risk factors such as hypertension, diabetes, and obesity. Obesity contributes to cardiac overload by increasing blood volume and peripheral resistance^(8)^. In younger individuals, DD is frequently underestimated due to the lack of overt clinical symptoms, emphasizing the need for early screening, especially among high-risk groups.

DD is closely linked to the development of HF with preserved ejection fraction (HFpEF), which accounts for approximately 50% of all HF cases and is associated with high rates of hospitalization and mortality-even in asymptomatic stages^(9)^. Additionally, DD is an independent predictor of adverse cardiovascular outcomes, including myocardial infarction, arrhythmias, and sudden cardiac death. Individuals with DD have up to three times the risk of progressing to HF, which significantly impacts their quality of life and functional capacity^(10)^.

The diagnosis of DD is primarily based on echocardiography, which assesses parameters such as the E/A ratio (from mitral inflow), the E/e’ ratio (myocardial relaxation index), and left atrial volume^(11)^. However, the technical complexity and cost of echocardiography limit its widespread use in primary care, creating barriers to the early detection of at-risk individuals. Furthermore, the scarcity of large-scale population studies on DD hinders efforts to characterize the condition in asymptomatic individuals within the community.

Early screening is critical for mitigating the impact of DD, enabling timely intervention during the initial stages of disease. Primary care plays a vital role in identifying risk factors, promoting health education, and facilitating referrals for specialized assessment.

The incorporation of point-of-care ultrasound (POCUS) into clinical practice has significantly broadened diagnostic and screening possibilities in primary care. POCUS is a portable, relatively low-cost, and user-friendly ultrasound modality that provides immediate, practical information at the bedside. For primary care nurses, POCUS offers a valuable tool for the initial screening of DD in asymptomatic patients, allowing for the assessment of basic parameters such as ventricular filling patterns and the presence of left ventricular (LV) hypertrophy. While less comprehensive than traditional echocardiography, POCUS can detect alterations suggestive of dysfunction that warrant further evaluation^(12)^.

Although echocardiography performed by cardiologists remains the gold standard for functional and structural cardiac assessment, the use of POCUS by nurses represents an opportunity to expand access to initial evaluations, particularly in the screening of DD among asymptomatic individuals. The implementation of POCUS by nurses not only optimizes the use of limited resources in settings with restricted access to cardiologists but also supports continuity of care by enabling ongoing monitoring of patients with cardiovascular risk factors. This approach is especially pertinent in low-income populations and remote regions where access to advanced diagnostic technologies is limited^(13)^.

A review of the MEDLINE/PubMed (Medical Literature Analysis and Retrieval System Online/Public Medical Literature), Cochrane Database of Systematic Reviews, and LILACS (Latin American and Caribbean Health Sciences Literature) databases found no prior studies that established an association between echocardiographic parameters indicative of cardiac dysfunction and prognosis in patients with or without symptoms of heart failure in the primary care context.

In this context, it is crucial to include cardiovascular sciences in the Sustainable Development Goals (SDGs), established in 2015 by the United Nations (UN) to be achieved by 2030^(14)^. Cardiovascular science can contribute to several targets, including “Good Health and Well-Being,” by supporting efforts to reduce premature mortality from non-communicable diseases such as cardiovascular disease, through prevention, early diagnosis, and effective treatment strategies^(15)^.

Health professionals can also play a key role in controlling risk factors such as hypertension, diabetes, obesity, and smoking through public health programs and clinical interventions. Physicians and nurses with specialized training are essential for expanding access to medical services and diagnostic technologies, including echocardiograms, POCUS, and stress tests, thereby enhancing cardiovascular care for populations worldwide.

## OBJECTIVES

To assess the potential of the ratio between early mitral inflow velocity and late diastolic mitral inflow velocity (E/A) as a prognostic factor for hospitalization due to cardiovascular causes and mortality in primary health care; and to identify the main NANDA-I nursing diagnoses^(16)^, interventions^(17)^, and outcomes^(18)^ associated with the medical diagnosis of DD in primary care.

## METHODS

### Ethical aspects

This study was approved by the Research Ethics Committee of the Fluminense Federal University School of Medicine. Written informed consent was obtained from all individuals who participated in the study.

### Study design

This study utilized data from the DIGITALIS Study, a longitudinal, community-based observational study designed to estimate the prevalence of different stages and phenotypes of HF in individuals enrolled in the Family Doctor Program (*Programa Médico de Família* - PMF) in the municipality of Niterói, Brazil. Data collection occurred between 2011 and 2012 (baseline, with face-to-face assessments), and between 2017 and 2018 (follow-up via telephone consultation and review of PMF medical records)^(19,20)^.

At baseline, 633 individuals aged 45 to 99 years were recruited and underwent clinical evaluations, including tissue Doppler echocardiography. After several years of follow-up, all participants were contacted and their PMF medical records were reviewed to determine the occurrence of hospitalizations and their causes, as well as mortality. All deaths were confirmed by either the Niterói Municipal Health Department or the Rio de Janeiro State Department of Health.

A population-based study showed a 7% reduction in the total incidence of HF between 2002 and 2014, primarily driven by decreased rates among individuals aged 60-84 years^(21)^. However, the incidence remained stable-or even increased-among younger patients (<55 years) and the very elderly (>85 years), groups that have historically received less clinical attention^(22)^.

### Population

Inclusion criteria were: active enrollment in the PMF of Niterói, Rio de Janeiro, Brazil; age between 45 and 99 years; the ability to attend the health unit on the scheduled date; and the ability to respond to the structured questionnaire.

Details regarding the sample size, selection criteria, recruitment process, and visit procedures have been published elsewhere^(23)^. All members of the research team underwent prior training.

Of the 633 individuals who completed the baseline evaluation, 73 were lost to follow-up (attrition rate = 11.5%). Thus, the final analytical sample comprised 560 participants. While interpretations may vary, it is generally accepted that losses below 5% lead to minimal bias, whereas losses above 20% may pose serious threats to internal validity^(24)^.

**Figure 1:**
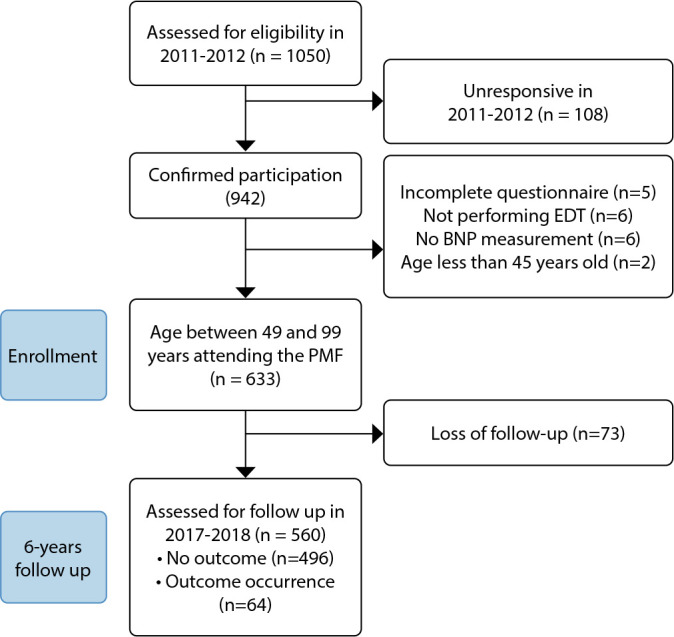
Recruitment flowchart based on the STROBE guideline

The recruitment flowchart based on the STROBE guideline is presented in the following Figure 1.

*STROBE flowchart by The Strengthening the Reporting of Observational Studies in Epidemiology (STROBE)*

The exploratory variable of interest was the transmitral flow velocity at the beginning of diastole (E) divided by the transmitral flow velocity at the end of diastole (A). Two distinct cut-off values were used, based on recognized guidelines^(11,24,25)^: Cut-off 1 - Grade I DD: ≤ 0.8; Grade II DD: > 0.8 and ≥ 2.0; Grade III DD: > 2.0. Cut-off 2 - Normal or Grade I DD: ≤ 0.75; Grade II DD: > 0.75 and ≤ 1.5; Grade III DD: > 1.5^(11)^.

The composite outcome was defined as hospital admission due to cardiovascular causes or death from any cause. The time between exposure and outcome was calculated as follows: a) for individuals who experienced the outcome, the time between the baseline evaluation and the occurrence of the event (hospitalization and/or death); b) for censored individuals, the interval between the baseline and the final follow-up assessment.

All echocardiographic examinations were performed in accordance with the recommendations of the American Society of Echocardiography (ASE) and the European Association of Echocardiography (EAE)^(25)^.

### Statistical analysis

Differences in the presence or absence of outcomes were examined using Pearson’s chi-square test with continuity correction, Fisher’s exact test, and, when appropriate, the Mann-Whitney U test. Crude hazard ratios were estimated for categorical echocardiographic parameters. Multivariable Cox regression models were constructed using variables associated with the outcome at a p-value threshold of 0.10 in univariable analyses. In the final model, a p-value < 0.05 was considered statistically significant. The adequacy of the multivariable Cox model was assessed using Schoenfeld residuals.

## RESULTS

For characterization of the study’s first phase, the distribution of HF stages was as follows (stage, n, percentage): 0 (78; 12.3%), A (248; 39.2%), B (243; 38.4%), and C (64; 10.1%). The second phase included 560 participants, of whom 355 (63%) were women. The mean age was 59.4 years, and 25% of the participants were older than 65 years. A total of 405 participants (72.3%) were diagnosed with hypertension, 133 (23.8%) with diabetes, and 99 (17.7%) were current smokers.

Table 1 presents the association between clinical variables and the composite outcome. Only age, systolic blood pressure (SBP), and pulse pressure were statistically associated with the outcome.

**Table 1:** Characterization of study participants according to the occurrence of the composite outcome

Variable	No outcome n= 496 n(%)	Outcome occurrence n= 64 n(%)
Age (years)^π^*^^	51-57-64.7	58-67-76.7
Female sex, n (%)^Ω^	321 (64.7)	34 (53.1)
Marital status: steady partners, n (%)^Ω^	325 (65.8)	41 (64.1)
Skin color: nonwhite, n (%)^Ω^	291 (5.3)	45 (70.3)
Education: ≥ 5^th^ grade n (%)^Ω^	290 (58.7)	31 (48.4)
Income: ≤ 1 minimum wage, n (%)^Ω^	369 (75.3)	47 (78.3)
Smoking: current smoking, n (%)^Ω^	85 (17.1)	14 (21.9)
SBP (mmHg)^π^*^^	120-133-147.5	128.5-143.7-168.5
DBP (mmHg)^π^	74.3-82-89.5	71.3-83.5-93.3
Pulse pressure (mmHg) ^π^*^^	43-51-60	48-63-84
HR (bpm)^π^	63-70.2-77	64-74-80.5
BMI (kg/m^2^) ^π^	24.5-27.2-30.6	23.6-26.7-31.5
Waist circumference (cm) ^π^	84-92-100	82-93-104

& Composite outcome: hospital admission due to cardiovascular causes or death from all causes. SBP: systolic blood pressure; DBP: diastolic blood pressure; HR: heart rate; BMI: body mass index; π: 1st quartile-median-3rd quartile; differences tested using the Mann-Whitney U test. Ω Differences tested using Pearson’s chi-square test;

* p-value < 0.05.

The association between echocardiographic findings and the composite outcome is presented in Table 2. At both cut-off points, the E/A ratio was significantly associated with the occurrence of the composite outcome.

**Table 2:** Crude hazard ratios for the occurrence of the composite outcome & according to echocardiographic changes in the ratio of early mitral inflow velocity to late diastolic mitral inflow velocity

Ratio of initial mitral flow velocity and end-diastolic mitral flow velocity (E/A, m/s)	Composite outcome^&^		Crude Hazard Ratio ^#^	95% CI
	Yes	No		
	n(%)	n(%)		
Cutoff 1^***^				
Grade I DD ≤0.8	21 (6.3)	314 (93.7)	1	
Grade II DD >0.8 and ≥2.0	40 (18.8)	173 (81.2)	3.47	2.00-6.02
Grade III DD >2.0	2 (18.2)	9 (81.9)	3.60	0.84-15.47
Cutoff 2^***^				
Normal or Grade I DD ≤ 0.75	24 (7.5)	297 (92.5)	1	
Grade II DD > 0.75 and ≤ 1.5	36(20.8)	137(79.2)	3.02	1.78-5.12
Grade III DD > 1.5	3(4.6)	62(95.4)	0.41	1.00-1.76
Grade III ≤140	5(22.7)	17(77.33)	2.31	0.92-5.77

& Composite outcome: hospital admission due to cardiovascular causes or death from all causes. #Estimated using Cox regression. Simple Cox regression p-values:

*** < 0.01.

In the DIGITALIS Study, the diagnosis of HF was established through a structured diagnostic questionnaire and/or considered in patients already receiving conventional pharmacological treatment. Importantly, 84.9% of participants presented alterations in the ratio of early mitral inflow velocity to late diastolic mitral inflow velocity despite not having a formal diagnosis of HF.

Table 3 presents hazard ratios (HRs) adjusted for age (continuous) and sex. All echocardiographic parameters with crude associations that reached statistical significance (p < 0.1) were included in the adjusted analysis.

**Table 3:** Adjusted hazard ratios for the occurrence of the composite outcome & according to echocardiographic changes in the ratio of early mitral inflow velocity to late diastolic mitral inflow velocity

Ratio of initial mitral flow velocity and end-diastolic mitral flow velocity (E/A, m/s)	Adjusted Hazard Ratio^#^	95% CI
Cutoff 1^*^		
Grade I DD ≤0.8	1	
Grade II DD >0.8 and ≥2.0	1.69	0.91-3.14
Grade III DD >2.0	2.80	0.64-12.31
Cutoff 2^*^		
Normal or Grade I DD ≤ 0.75		
Grade II DD > 0.75 and ≤ 1.5	1	
Grade III DD > 1.5	1.53	0.85-2.73
Grade III ≤140	0.49	0.11-2.10

& Composite outcome: hospital admission due to cardiovascular causes or death from all causes. # Estimated by multiple COX regression including continuous age and sex, in addition to each echocardiogram parameter. Multivariable Cox regression p value:

*** <0.1.

The associations between the composite outcome and the E/A ratio were not considered statistically significant, as the p-values did not reach the < 0.05 threshold. In all cases, a reduction in the strength of associations was observed after adjustment, indicating that age or sex acted as moderate confounders in the relationship between the echocardiographic parameters and the outcome.

Chart 1 presents the Nursing Diagnoses (NANDA-I), the corresponding Nursing Interventions (NIC) applied to these patients, and the expected Nursing Outcomes (NOC) for each diagnosis.

**Chart 1:** Nursing diagnosis (NANDA-I), Interventions (NIC) and Outcomes (NOC) for patients with diastolic dysfunction.

DIAGNOSIS	DEFINITION	DEFINING CHARACTERISTICS/RISK FACTORS
Activity Intolerance (00092)	Insufficient physiological or psychological energy to support or complete necessary or desired activities	Dyspnea, fatigue, and generalized weakness after minimal exertion
Ineffective tissue perfusion (00204)	Reduction of oxygen resulting in failure to nourish tissues at the capillary level	Cold extremities, pallor, reduced exercise capacity
Fatigue (00093)	Sustained and overwhelming feeling of exhaustion and reduced capacity for physical and mental work	Decline in functional performance and decreased ability to maintain daily routines
Deficient knowledge (00126)	Deficiency or lack of information related to health and clinical condition	Frequently asked questions about the condition, inconsistent or incorrect information about diastolic dysfunction
Anxiety (00146)	Vague and uncomfortable feeling of apprehension or fear accompanied by an autonomic response	Health-related worry, muscle tension and insomnia
Impaired sleep pattern (00198)	Prolonged interruption in the quantity and quality of sleep due to physical or emotional factors	Report of difficulty initiating or maintaining sleep, fatigue on waking
Risk of decreased cardiac output (00240)	Susceptibility to cardiac pumping of insufficient volume to meet metabolic demands in individuals with cardiovascular and/or pulmonary problems or trauma	Pre-existing heart disease
Risk of unbalanced blood pressure (00362)	Susceptibility to a recurrent increase or decrease in the force exerted by the blood flow on the arterial wall, above or below the desired individual levels	Cardiovascular diseases and the use of medication
**NIC**	**DEFINITION**	**ACTIVITIES**
Cardiovascular health education (5510)	Educate patients about modifiable risk factors, such as diet, exercise, weight control and adherence to drug therapy	Explain the warning signs of cardiac decompensation; Provide educational materials adapted to the patient’s level of understanding.
Activity control (0180)	Establish activity limits based on individual tolerance	Encourage rest breaks during daily activities; Propose light physical activity, such as monitored walking, to improve functional capacity.
Hemodynamic monitoring (4250)	Assess vital signs, especially heart rate and blood pressure	Observe for signs of worsening perfusion, such as cold extremities and cyanotic skin; Regularly assess body weight to monitor water retention.
Self-care promotion (1800)	Involving the patient in care planning	Provide guidance on preventive measures to avoid complications; Develop a personalized self-care plan, including home blood pressure monitoring.
Anxiety management (5820)	Offer emotional support and coping strategies to reduce stress	Guide relaxation techniques, such as deep breathing and guided meditation; Facilitate access to support groups for patients with cardiovascular conditions.
Improving sleep patterns (1850)	Guidance on sleep hygiene, including a regular bedtime routine and avoiding stimulants at night	Assess the need for adjustments to the sleep environment to increase comfort; Monitor the impact of medication on sleep quality.
**NOC**	**INDICES**	**TARGET**
Physical endurance (0008)	Increased ability to perform daily activities without excessive fatigue	Reduce the intensity and frequency of dyspnea episodes
Knowledge: controlling heart disease (1842)	Demonstration of knowledge about the condition and preventive measures	Increasing adherence to therapeutic guidelines
Energy level (0002)	Improved perception of energy for daily activities	Promoting self-sufficiency in activities of daily living
Cardiovascular status (0402)	Heart rate and blood pressure within desirable limits	Maintaining hemodynamic stability
Reducing anxiety (1402)	Reported lower frequency of worrying thoughts	Improve emotional well-being and reduce the impact of stress
Improved sleep pattern (0004)	Reported longer and better-quality sleep	Promote adequate physical and mental recovery

## DISCUSSION

Changes in the E/A ratio indicate impaired diastolic function and are associated with a worse prognosis^(26)^. The associations of this parameter with the outcome were initially strong and statistically significant. However, after adjustment for age and sex, the association between the composite outcome and the E/A ratio was no longer significant, suggesting that the sample may have been underpowered for this analysis. In a study involving 3,600 middle-aged and older individuals from Indian communities, Bella et al.^(27)^ demonstrated that an E/A ratio >1.5 m/s was independently associated with a threefold increase in cardiac mortality.

Several authors have observed that mitral inflow wave velocity, represented by the E/A ratio, may have prognostic implications in the presence of systolic dysfunction. Patients who presented with an increased E/A ratio and markedly shortened deceleration time (DT), characterizing a restrictive filling pattern, had a higher risk of cardiovascular morbidity and mortality. These two diastolic parameters may therefore serve as predictors of poor prognosis in HF^(28)^. A restrictive LV filling pattern is an independent predictor of mortality in patients with non-ischemic dilated cardiomyopathy, conferring more than a threefold increase in the risk of death (adjusted HR 3.2, 95% CI 1.8-5.7; p = 0.03)^(29)^.

Nagueh et al. described that the combination of a high E/A ratio and left atrial enlargement, even in the presence of preserved ejection fraction, characterizes a restrictive filling pattern and is comparable to the poor prognosis associated with the classic restrictive phenotype. Since the E/A ratio is reproducible and easy to obtain, it facilitates the identification of different patterns of DD (grades I, II, or III) and their correlation with LV filling pressures, offering both diagnostic and prognostic value, as well as informing functional classification^(11)^.

The DIGITALIS study also identified individuals at high risk of cardiovascular hospitalization and all-cause mortality. When adjusted for sex and age over a six-year follow-up period, the associations did not reach statistical significance. In this study, the E/A ratio was abnormal in 84.9% of participants without a formal diagnosis of HF, indicating the presence of subclinical HF (stage B). Our findings corroborate those of the DIGITALIS study, showing that individuals with risk factors such as advanced age, hypertension, diabetes mellitus, and obesity-and who demonstrated DD classified as grade II or III based on the E/A ratio-were associated with adverse cardiovascular outcomes^(30)^.

In a six-year retrospective cohort study of 36,261 individuals, Halley et al. found that moderate and severe DD were associated with an increased risk of all-cause mortality, with HRs of 1.58 (grade II) and 1.84 (grade III), respectively (p < 0.01)^(26)^. These results are consistent with the present study involving 633 individuals and the same follow-up period.

The management of DD in primary care requires a comprehensive approach that encompasses both clinical and educational strategies. Nursing diagnoses such as activity intolerance and deficient knowledge highlight key areas where nursing interventions can improve patient outcomes. Implementing actions such as cardiovascular health education and self-care promotion empowers patients to take an active role in managing their condition. Outcomes such as improved physical endurance and cardiovascular status provide measurable indicators of the impact of the care delivered^(16,17)^.

In addition, adopting evidence-based practices ensures that interventions are aligned with the most current guidelines, thereby providing more effective care. Strategies such as continuous education for healthcare professionals and regular monitoring of patients’ conditions are essential for identifying early signs of deterioration and adjusting the care plan as needed. The integration of technologies, such as health monitoring apps and telemedicine, can optimize follow-up and enhance patient engagement in self-care^(31)^.

Another important aspect is the management of anxiety and sleep disorders, which are often associated with DD. These factors can worsen symptoms and hinder treatment adherence. Therefore, strategies such as emotional support, guidance on relaxation techniques, and interventions to improve sleep patterns are indispensable for a comprehensive care approach^(32)^.

Finally, coordination among multiprofessional teams is essential to ensure comprehensive care. Nurses, physicians, nutritionists, and physiotherapists must work collaboratively to plan and implement individualized management strategies that consider each patient’s specific needs. The implementation of public health policies that promote heart disease prevention and access to affordable treatments is crucial for reducing health disparities.

### Limitations

The echocardiographic examinations were conducted using four portable devices from different manufacturers, all of which were calibrated, properly maintained, and in full working order. However, the recordings could not be stored on backup devices, which prevented the calculation of the intraclass correlation coefficient for intraand inter-examiner variability.

### Clinical applicability

The use of transthoracic echocardiography with Doppler in primary care may enable the identification of individuals who would benefit most from this investigation-specifically, those in stages B and C of HF-thus aiding in the exclusion or confirmation of HF in asymptomatic and/or symptomatic individuals.

The combination of conventional echocardiography performed by physicians and POCUS conducted by nurses in primary care represents a significant advancement in healthcare, particularly for screening asymptomatic patients with DD. Despite existing challenges, this integration broadens access to early diagnosis, strengthens the role of nursing professionals, and promotes a collaborative care model. Successful implementation depends on adequate training, the development of standardized protocols, and the incorporation of technology to support clinical practice.

## CONCLUSION

Primary care is a critical setting for managing patients with DD, requiring the application of evidence-based nursing diagnoses, interventions, and outcomes. A patient-centered approach, combined with technical and scientific knowledge, can significantly improve the quality of life of affected individuals.

Most participants with alterations in the echocardiographic E/A ratio had not been diagnosed with HF, suggesting they may be classified as stage B HF and could benefit from therapeutic interventions aimed at reversing cardiac changes.

By evaluating the E/A ratio - a parameter of diastolic function - this study identified individuals at high risk of cardiovascular hospitalization and all-cause mortality in the primary care setting, underscoring the importance of incorporating this examination into routine care.

Cardiovascular sciences play a vital role in promoting global health and reducing social inequalities, particularly in the context of cardiovascular disease. By contributing to prevention and treatment, cardiology supports Sustainable Development Goals related to health, equity, and innovation. Achieving these goals requires collaboration among healthcare professionals, policymakers, and international organizations to implement effective public policies and innovative solutions against heart disease.

## Data Availability

Research data is only available upon request.
